# Fogging with Hydrogen Peroxide and Hypochlorous Acid: An Option for Disinfection and Reuse of Disposable Isolation Gowns in Medical Practice

**DOI:** 10.3390/microorganisms13071537

**Published:** 2025-06-30

**Authors:** Shay Iyer, Zenhwa Ouyang, Arathi Vinayak

**Affiliations:** 1 VCA West Coast Specialty and Emergency Animal Hospital, 18300 Euclid Street, Fountain Valley, CA 92708, USA; shay.iyer@gmail.com; 2 1 IDEXX Drive, Westbrook, ME 04098, USA; ouyang1234@gmail.com

**Keywords:** disinfection, fogger, isolation gowns, sterilization, microbes, PPE, validation

## Abstract

A total of 1.6 million tons of personal protective equipment (PPE) waste has been generated daily since 2019 and this production has not abated since that time. Within PPEs, isolation gowns make up the largest percentage by weight of landfill waste. This study aimed to evaluate the effectiveness of rapid, reproducible disinfection protocols to help facilitate safe reuse and minimize risks from microbial contamination. Disinfection of isolation gowns via fogging with hydrogen peroxide (HP) and hypochlorous acid (HC) were evaluated in the present study compared to standard ethylene oxide (EO) sterilization. This study was conducted at VCA West Coast Specialty and Emergency Animal Hospital in the United States. Ten isolation gowns (control) were cultured on tryptic soy agar contact plates in 10 predetermined areas to determine microbial load and morphology/types on non-sterile gowns before use. Following this, 10 gowns were fogged with 12% HP, and then once drying was complete, they were cultured in the predetermined areas for microbial load and morphology/types. This procedure was repeated with another set of 10 gowns fogged with 500 ppm HC. Lastly, 10 gowns were sterilized with EO using standard protocol and cultures were performed similarly. Median CFU (colony-forming unit) counts at 48 h for control, EO, HP, and HC were 4.5, 0, 0, and 0; at 72 h, they were 107, 0, 0, and 0, respectively. No significant difference was noted between the disinfection groups; post hoc pairwise analysis showed that the CFU counts for the disinfection groups were significantly lower than those for the control. The median percent reduction at 48 h for EO, HP, and HC was 100, 100, and 100; at 72 h, it was 100, 100, and 100, respectively. No significant difference was detected among the groups. The median number of microbe types for control, EO, HP, and HC was 2.5, 0, 0, and 0; there was no difference between the disinfection groups, but the number of microbe types was significantly higher for the control than for the disinfection groups. EO is environmentally toxic, expensive, and carcinogenic; it requires prolonged disinfection cycle times, expensive equipment, and trained personnel. This study suggests that HP and HC provide a cost-effective, relatively nontoxic, environmentally safe, and comparatively short disinfection time option for the disinfection and reuse of isolation gowns that does not require trained personnel or specialized equipment.

## 1. Introduction

Disinfection is defined as a process that eliminates most if not all pathogens on inanimate objects, the exception being endospores [1]. Sterilization is the process that eliminates all microbes including spores by physical or chemical methods [2]. Pathogens are capable of surviving on inanimate surfaces for a period of hours to years and this in turn can lead to further transmission. This has led to stringent disinfection protocols for surfaces and a push for single-use PPE, which consists of items of clothing worn by workers in healthcare to minimize the risk of transfer of solid or liquid pathogens. PPE consists of gloves, masks, hand sanitizers, and gowns aimed at covering the torso, arms/hands, and legs. Gowns fall into two broad categories: isolation and surgical. Surgical gowns are sterile and reserved for high-fluid-level situations, whereas isolation gowns are used for non-sterile patient care. They differ in material composition and fabric weight. Isolation gowns are used in patient-facing roles for contagious infections, as well as in nursing homes and oncology and dialysis clinics.

Single-use, disposable isolation gowns are the greatest percentage of landfilled PPE by weight (85%) [3] and are mostly manufactured using non-degradable synthetic materials like polypropylene, spunbound–meltdown–spunbound (SMS), or polyethylene-coated non-woven material [4]. A study determined that an average of 33 isolation gowns per day per patient were used for stable patients, and the number of gowns was much higher in unstable patients [5]. In 2022, 2.5 billion gowns were purchased in the United States [5] and there has been a 2000% price increase in the price of isolation gowns [6].

The consequence of landfilling isolation gowns is multifold: 1. Their continued production, as well as lack of degradation, has led to a bioburden on landfills and overflow into oceans and the environment [7,8]. 2. Microbes on isolation gowns have the potential to contaminate ground water and pollute the air, with the potential to disseminate harmful pathogens [9]. 3. The degradation of plastics from isolation gowns into micro- and nanoplastics has endangered living organisms and is being implicated in non-communicable disease in humans [10,11,12]. 4. Incineration of isolation gowns/PPEs leads to the release of toxic gases and continued need to landfill ash [4,13]. These drastic consequences from landfilling gowns warrant immediate attention. Solutions need to focus on increasing the reuse of isolation gowns/PPE via simple but effective disinfection methods.

List Q of the Environmental Protection Agency (EPA) provides a list of disinfectants that are labeled for use against emerging pathogens [14]. This list was extensively researched for disinfectants with a high safety profile and cross-referenced with the CDC-approved list of chemical disinfectants [15]. There are numerous studies evaluating the efficacy of hydrogen peroxide (HP) both by itself in aerosolized and vapor forms for sterilization/disinfection [16,17,18,19] and in combination with ozone [20]. HP is considered both a disinfectant and a sterilant, depending on how it is utilized. Methods such as HP vapor (HPV), vaporized HP (VHP), and HP gas plasma (HPGP) are considered sterilization methods, as they utilize high concentrations of HP, which are sporicidal. In contrast, methods such as aerosolized HP (aHP) and HP fogging, where lower concentrations of HP are used, are considered to be disinfection, as they are variable in their efficacy against spores. The minimum effective concentration for HP recommended by the CDC for disinfection is 6–7.5% [15], while concentrations greater than 30% require very careful handling due to corrosiveness [15]. One study documented the efficacy of HP against Gram-positive organisms at concentrations of just 3% [21]. More commonly, commercially available HP is available in a 12% solution and was chosen for this study as it meets the minimum required concentration. There are numerous publications on the efficacy of hypochlorous acid (HC) for disinfection in gaseous and fog forms [22,23,24]. The minimum effective concentration needed to decontaminate insert surfaces is 200 PPM [25]. Commercially available solutions of HC are usually 500 PPM, which was used in this study in an undiluted form, as it meets the minimum effective concentration.

HP and HC were chosen for their established efficacy, low toxicity, ready availability for purchase, and inexpensiveness [14,15]. We chose to use a commercially available fogger to dispense these disinfectants rather than specialized equipment to produce vapor forms under pressure or gaseous forms to determine whether inexpensive, easily available fogging equipment could reduce microbial loads on isolation gowns to levels safe for reuse. A fogger is an inexpensive machine that takes a solution and creates mist with small particle sizes less than 20–25 µm in diameter. When smaller particles are generated, there is a greater chance of suspension in the air, increasing the chances of the solution encountering the pathogen in the air, as well as ensuring uniform rapid coverage of the area to be disinfected. The ideal fogger should generate particle sizes less than or equal to 20 µm; the reported size range is from submicrometer to 200µm [26]. Inexpensive disinfectants with an easily replicable methodology are more likely to be utilized in daily medical practice. A more cumbersome methodology with toxic disinfectants is less likely to have compliance.

An “ideal” biocide is defined as an agent that is easy to use, easy to store, safe, has a long-lasting effect, and is environmentally friendly, with a chemical composition that is compatible with the surface it is being applied to [27]. The goals of this study are to compare two environmentally safe chemical disinfection agents that are ideal biocides (HP and HC) against gold-standard ethylene oxide (EO) sterilization. While HP and HC are disinfectants in the manner they are utilized in this study, EO is a sterilant. While a sterilant and disinfectant are different by definition, in this study, they serve the same purpose, which is to eliminate microbes.

We chose to compare HP and HC to EO as the latter is used extensively in hospitals for the sterilization of supplies and equipment. While there are numerous studies evaluating the superior efficacy of EO, there are also studies documenting its environmental pollution [28] and mutagenic, carcinogenic, and teratogenic potential, in addition to being implicated in osteoarthritis [28], sleep apnea [29], and liver dysfunction [30]. There are an estimated 10,000 EO sterilizers in the United States alone, and approximately 75,000 workers are exposed to EO in those facilities [31]. We chose to compare HP and HC to EO to determine whether environmentally safe, low-toxicity disinfectants perform similar to EO against resident microflora on gowns.

The primary objectives are to determine whether fogging with these disinfectants results in a rapid turnaround time for reuse, whether single-use isolation medical gowns can be safely reused using these disinfectants, and which product(s) disinfects isolation gowns most effectively. Our hypothesis is that the disinfection of single-use isolation gowns with HP and HC fogging will result in a reduction in the microbial count and types similar to EO sterilization. The hope is that with inexpensive disinfectants, a rapid technique, and an easily instituted way of disinfecting gowns, there is likely to be a higher compliance resulting in safe reuse and a reduction in the bioburden on landfills while safely protecting the personnel donning the gowns and those they are in contact with.

## 2. Materials and Methods

### 2.1. Methodology

Contact plates of tryptic soy agar (TSA) (Cole-Palmer, Vernon Hills, IL, USA) were chosen for all cultures in this study with neutralizing agents of lecithin and tween 80. TSA was chosen as a general-purpose non-selective medium. Prior to culture, refrigerated contact plates were warmed for 30 min in an incubator at 37 °C to increase organism recovery [32]. 

Four groups were evaluated in this study, each consisting of 10 unused gowns (10 control, 10 HP, 10 HC, and 10 EO).

**Figure 1:**
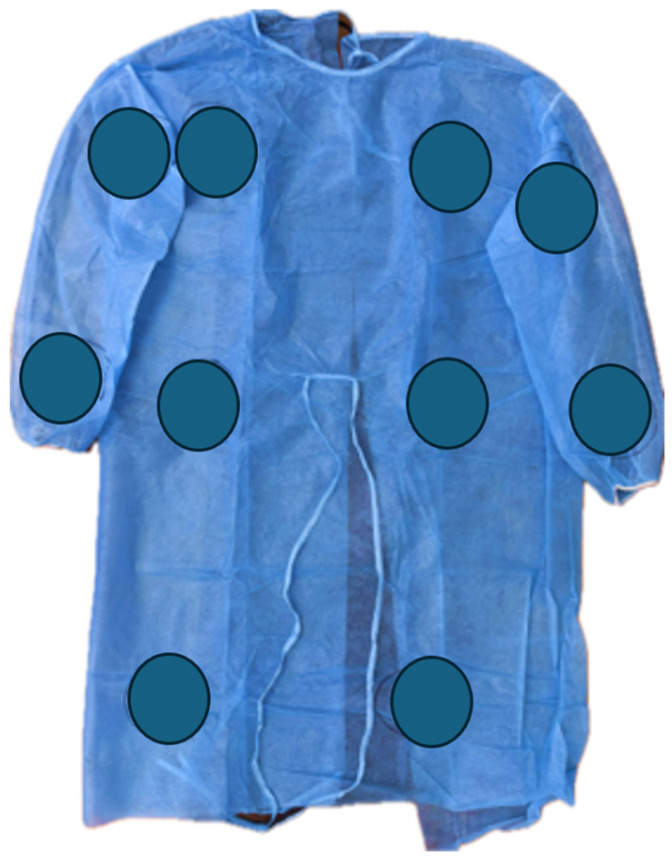
Image of an isolation gown with the predetermined areas to be cultured marked in circles.

A control group (Group 1) consisting of ten unused isolation gowns from the packaging were cultured using a single plate per gown along the arms (4 contact points), chest and body (4 contact points), and thigh portion of the gown (2 contact points) (Figure 1). None of the gowns in the study were inoculated with specific pathogens (fungi, viruses, or bacteria).

To minimize the contamination of the plates, gloves and a respirator mask were donned prior to handling the plates. The plates were pressed to the areas being sampled by firmly pressing the convex surface in a counterclockwise manner at each contact point. Agar plates were labeled as 1–10 to represent the 10 gowns in the group. The goal was to mimic the recovery of human pathogens in clinical practice; thus, incubation of the agar plates was performed at 37 °C, similar to temperatures in the human body (35–37 °C), and the CFU (colony-forming unit)/plate counts were recorded at 48 h and 72 h into incubation. In addition, morphology at 72 h, which included microbial form, elevation, surface, opacity, pigment, and margin, was recorded. Digital images were obtained at each of the time points. CFU/plate counts were counted manually by two observers per plate.

**Figure 2:**
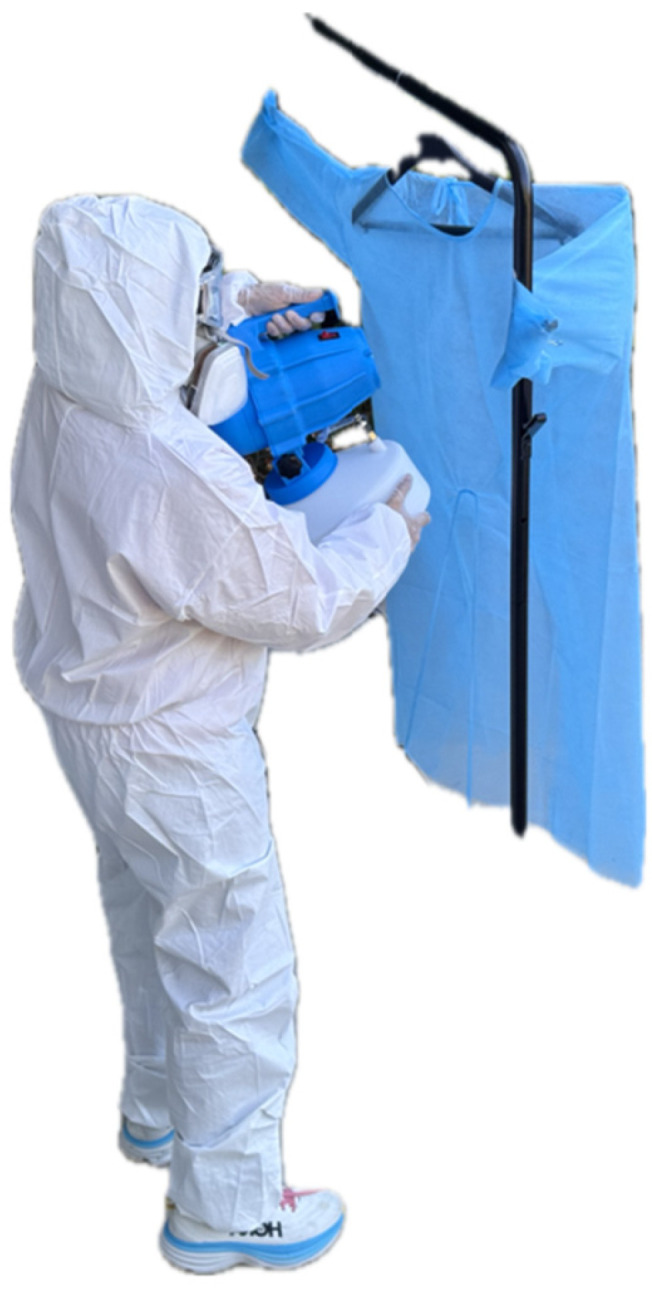
Fogging being performed on isolation gowns.

Ten gowns were then fogged with 12% (120,000 PPM) HP (SimpleNature (brand), Pacific Innovators (manufacturer), Elk Grove Village, IL, USA) (Group 2), after donning coveralls, a respirator, gloves, goggles, and shoe covers (Figure 2).

A fogger (Ceed4U, China) that generated particle sizes of 15–20 µm, to penetrate the texture of the disposable gowns and for a more even coverage of the gowns, was used to dispense the chemical. All fogging took place outdoors in a shaded area to limit ultraviolet light exposure and the potential deactivation of chemicals. Fogging was performed outdoors rather than indoors to prevent the continued exposure of humans indoors to disinfectants (HP and HC) and prevent the use of further disinfectants to remove HP and HC. Once fogging was completed, gowns were brought back indoors for an additional 4 h of drying time. Contact plates were then used to culture the gowns along the arms, thighs, and chest, similar to the control group. CFU/plate counts, as well as microbial characteristics to determine types, were recorded, similar to the control group. Digital images were obtained similar to group 1 at both time points into incubation. The fogging system was then cleaned out with mild detergent and copious water, as well as a run time with water alone for 5 min, prior to use with HC.

The procedure was repeated using the fogger filled with 500 PPM HC (Aquaclense (brand), Aqua Science (manufacturer), Newark, DE, USA) (Group 3) for another 10 gowns after the fogger. Methods similar to HP were utilized to fog the gowns and allow for disinfection. Cultures were performed in a similar manner to Groups 1 and 2.

Lastly, 10 gowns (Group 4) were sterilized using an EO sterilizer (Andersen EOGas Sterilizer AN333, and Andersen Abator AN5200, Haw River, NC, USA) using standard techniques [33]. The 5 stages of EO sterilization were instituted, including preconditioning and humidification, EO gas introduction, exposure, evacuation, and aeration. The EO sterilizer was used as an integrated aeration built into the cycle. The cycle length was 16 h, including aeration time. Mechanical aeration allowed for the desorption of the toxic residues. CFU/plate counts, as well as microbial characteristics, were recorded, similar to the control group.

### 2.2. Statistical Analysis

Intraclass correlation coefficient (ICC) was used to assess the agreement between the CFU counts of Reader 1 and Reader 2. The CFU counts of Reader 1 (SI) and Reader 2 (AV) were averaged if the ICC was 0.9 or more and statistical significance was detected (*p* < 0.05).

The percentage reduction in CFU counts was calculated for each plate in the EO, HP, and HC groups. The number of CFU counts per plate in a certain treatment group at a certain follow-up time was divided by the average number of CFU counts across all control plates at the same follow-up time and multiplied by 100.

Boxplots were created to visually compare the CFU counts, percent reduction across disinfectant methods, and number of microbe types. CFU counts, percent reductions, and number of microbe types lying 1.5 × IQR (interquartile range) below the first quartile or above the fourth quartile were classified as outliers. The Kruskal–Wallis test was used to detect significant differences among the treatment groups. Dunn’s test was used to detect pairwise differences using Bonferroni’s correction for multiple comparisons.

## 3. Results

**Figure 3:**
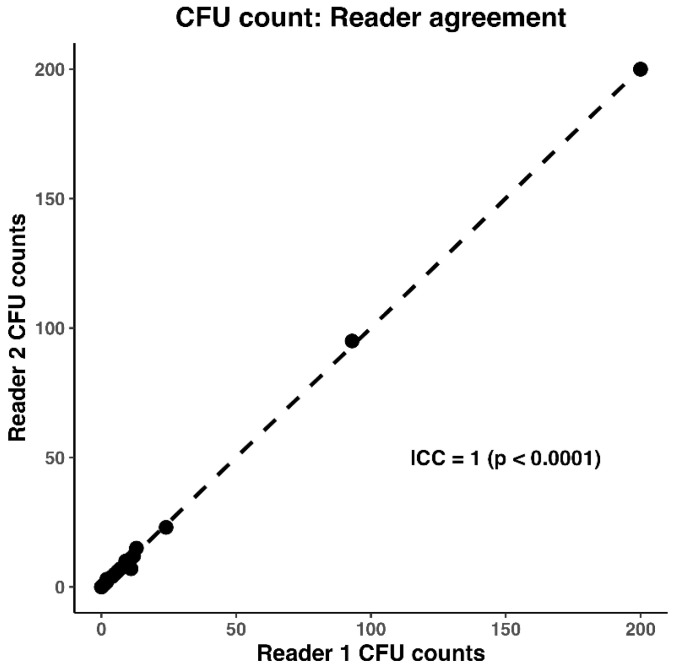
Intraclass correlation coefficient (ICC) of 1, showing there is strong agreement between the CFU counts of Reader 1 (SI) and Reader 2 (AV).

Statistical analysis was run using RStudio 2024.09.0+375 (R Core Team 2024). R: A Language and Environment for Statistical Computing (R Foundation for Statistical Computing, Vienna, Austria, https://www.R-project.org/). Since the ICC was 1 (*p* < 0.0001) (Figure 3), the two reads for each plate were averaged into a single value.

**Figure 4:**
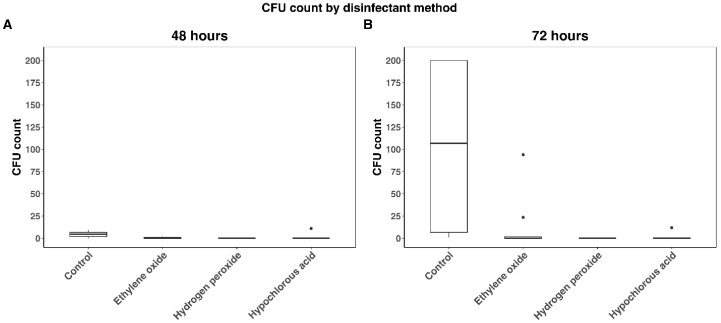
CFU counts by disinfectant method at 48 and 72 h. (**A**) Represents CFU at 48 h by disinfection method. (**B**) Represents CFU at 72 h by disinfection method. Black dots indicate outliers and were defined as 1.5 × IQR (interquartile range) below the first quartile or above the fourth quartile. CFU counts were lower for ethylene oxide, hydrogen peroxide and hypochlorous acid than controls (*p* < 0.0001) at both timepoints. No significant differences were detected among ethylene oxide, hydrogen peroxide and hypochlorous acid at either timepoint.

Median CFU counts at 48 h for the control, EO, HP, and HC groups were 4.5 (IQR: 2–6.75; mean: 4.65), 0 (IQR: 0–1; mean: 0.6), 0 (IQR: 0–0; mean: 0), and 0 (IQR: 0–0; mean: 1.1), respectively. At 72 h, the median CFU counts were 107 (IQR: 6.75–200; mean: 103.25), 0 (IQR: 0–1.75; mean: 12.05), 0 (IQR: 0–0; mean: 0), and 0 (IQR: 0–0.75; mean: 1.4), respectively. The Kruskal–Wallis test detected a significant difference among CFU counts at 48 and 72 h (*p* < 0.0001) (Figure 4).

Post hoc pairwise comparisons using Dunn’s test found that CFU counts for EO, HP, and HC were significantly lower than those of controls (Table 1), though no differences were detected among the non-control treatment groups.

**Figure 5:**
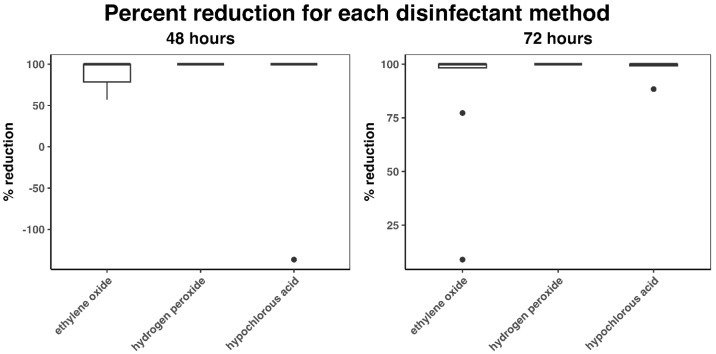
Percent reduction in CFU count (compared to control) at 48 and 72 h. Black dots indicate outliers and were defined as 1.5 × IQR (interquartile range) below the first quartile or above the fourth quartile.

Median percent reductions at 48 h for the EO, HP, and HC groups were 100 (IQR: 78.5–100; mean: 87.1), 100 (IQR: 100–100; mean: 100), and 100 (IQR: 100–100; mean: 76.34), respectively (Figure 5). At 72 h, median percent reductions were 100 (IQR: 98.31–100; mean: 88.33), 100 (IQR: 100–100; mean: 100), and 100 (IQR: 99.27–100; mean: 98.64), respectively. The Kruskal–Wallis test (*p* = 0.07) did not detect any significant differences among the groups.

One outlier was detected for HC at 48 h (Figure 4 and Figure 5). Two outliers for EO and one for HC were detected at 72 h. The two outliers detected for HC (one at 48 h and one at 72 h) occurred on the same plate.

**Figure 6:**
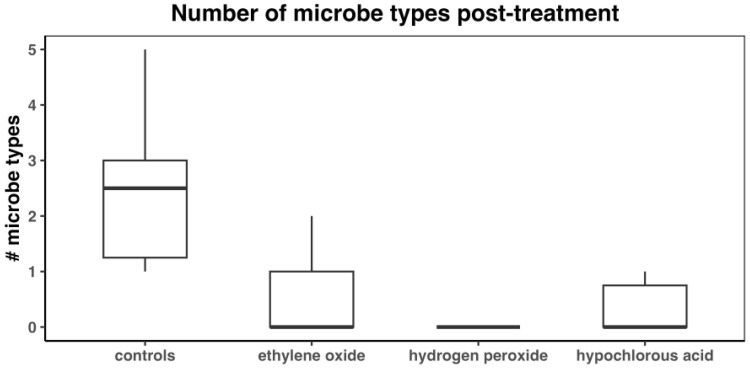
Number of microbe types after treatment with disinfectant.

The median numbers of microbe types for controls and the EO, HP, and HC treatment groups were 2.5 (IQR: 1.25–3; mean: 2.4), 0 (IQR: 0–1; mean: 0.5), 0 (IQR:0–0; mean: 0), and 0 (IQR: 0–0.75; mean: 0.3) (Figure 6). The Kruskal–Wallis test detected a significant difference among the treatment groups (*p* < 0.0001). The number of microbe types in the control group was significantly higher than that in the other treatment groups (Table 2). No differences between EO, HP, and HC were detected.

Fogging times were recorded from five randomly chosen gowns in the HP and HC groups (Table 3). Mean fogging times were calculated for the HP and HC groups. The mean for the HP group was 1 min:10 s. The mean for the HC group was 1 min:11 s.

## 4. Discussion

The results of our study show that median CFU counts at 48 and 72 h were significantly lower in the HP, HC, and EO groups when compared to the control. Statistically there was no difference in this reduction between the disinfection groups. Similarly, the median percent reduction in microbial levels at 48 and 72 h, as well as microbial types at 72 h, for the HP, HC, and EO groups was significantly different compared to the control group, and there were no differences in this reduction between disinfection groups. The total time required to fog isolation gowns was similar across the HP and HC groups, with a mean 1 min:10 sec for the HP group and 1 min:11 sec for the HC group. Even with the 4 h drying time after fogging that we arbitrarily chose, the process was relatively short when compared to EO sterilization. We chose 4 h to allow for particles in the air to settle and allow for adequate drying time (all gowns were fully dry at the time of cultures). EO time cycle times, which include sterilization and aeration times, range from 10.5 to 14.5 h [2].

EO has emerged as the preferred sterilization method for medical devices, effectively targeting bacteria, viruses and spores [34]. As a potent alkylating agent, it inactivates cellular components like nucleic acid and functional proteins, resulting in the denaturation of microbes. Its advantages include rapid action, material permeability and compatibility, resilience against organic debris, and high efficacy [34]. Drawbacks include lengthy cycles, higher cost, toxicity, and carcinogenic risks [35].

We selected nontoxic, environmentally safe disinfectants in a simple and swift disinfection protocol without carcinogenic risks. This potentially increases compliance for disinfection and reusing isolation gowns in medical practice. The vapor form of HP is a highly effective, broad-spectrum biocide and, as per studies, is deemed ideal due to its proven efficacy against bacteria/fungi/viruses while offering superior human and environmental safety, ease of use, stability, and material compatibility [19]. HP has minimal environmental footprint as it decomposes into water and oxygen, leaving no surface residues. Its exact mechanism of action remains unknown, despite its documented biocidal action. Its proposed mechanism of action is the deactivation of microorganisms through the oxidation of macromolecules such as lipids, carbohydrates, proteins, and nucleic acids [27]. There are studies documenting the efficacy of HP on medical textiles [21,36,37], with many studies documenting its efficacy when used as a fog [38,39,40,41], but none regarding its use for the disinfection of isolation gowns.

HC is highly effective against a broad range of microorganisms by selectively binding to the unsaturated lipid layer, disrupting its integrity. It destroys viruses by forming chloramines and nitrogen-centered radicals, leading to DNA breaks and rendering the virus harmless [41]. There are numerous studies documenting its superior efficacy when delivered as a fog [22,23,42,43]. At concentrations >10% of HP, there are reports of skin, ocular, and mucous membrane irritation [44]. Some irritation to the eyes and the nasal passages during 12% HP fogging, despite the use of PPE including a respirator and goggles, were noted during this study, making HC a safer choice, as no irritation was noted. In other aspects of cost and procurement, both disinfectants are similar. At concentrations of 200 ppm, HC effectively disinfects surfaces with a 1 min contact time. The solution of HC we used decomposes to NaCl and water, which are safe for the environment and are not considered toxic residues to humans [15]. Free chlorine during HC fogging has the potential for adverse human effects. However, in a study evaluating free chlorine levels during HC fogging at concentrations of between 300 and 500 PPM, such as those used in this study, chlorine levels did not exceed the permissible level of 0.51 ppm [43]. Only at concentrations of HC greater than 2000 ppm was the free chlorine level found to be higher than the permissible level [43].

Contact plates, agar plates with a convex surface shape, allow for the culture of surfaces by pressing the plates against the surface to be cultured. They are primarily used to evaluate the microbial contamination of surfaces following disinfection. The convex shape allows for maximal contact and transfer of bacteria on to the surface of the plate. Though cotton swabs are often used to sample hospital environmental surfaces for cultures, contact plates are recommended for direct surface sampling and are widely accepted for accurately assessing the contamination levels in hospitals [45]. Contact plate methods have been shown to have better recovery of bacteria, especially in studies evaluating multidrug-resistant bacteria [46,47]. A recent study found contact plates outperformed swabs in detecting surface contamination on medical fabrics, isolating more microbial species [45]. In a study evaluating methods of the recovery of drug-resistant *Staphylococcus* from hospital environmental surfaces, the sampling method (swab vs. direct contact cultures) was more important than the choice of agar medium [46]. Neutralizing agents, like lecithin, azolectin, polysorbate 80, tween 80, sodium thiosulfate, and sodium bisulfite, in agar are essential post-disinfection to inactivate residual disinfectants, enabling microbe detection and efficacy assessment when surfaces are cultured [48]. We chose TSA as a non-selective agar with neutralizing agents of tween 80 and lecithin. Most contact plates have a printed grid pattern on the plates themselves or the agar to help with the counting of colonies across the plate. Dilutions for CFU/mL calculations are not performed with contact plates and the total count is reported as a CFU count per plate. It represents the number of bacteria from the surface sampled. If colonies are too dense to count, the result is reported as 200+ and “lawn” if the colonies coalesce. We have followed these guidelines in this study.

A limitation of this study, as with any disinfection study utilizing cultures, is the risk of contamination. While we have taken precautions to prevent contamination with gloves and masks when cultures were obtained, contamination from microbes in the air is a possibility. This would be true across the four groups. Another limitation is that genetic analysis was not performed to know which organism types were in each group and whether this changed following disinfection compared to the control group. While this information would have been interesting, it does not diminish the efficacy of HP and HC in this study, as evidenced by the reduction in CFU/plate or the decrease in the types of microbes noted. We did not test efficacy against specific pathogens (viruses, bacteria, or fungi) to know how HP and HC perform in those conditions. Fogging outdoors, despite being performed in the shade, could have led to UV-induced degradation of HP and HC. There was a rather high microbial bioburden in the control group. It would be interesting to see whether this high microbial bioburden was unique to the brand of gowns purchased for this study or true across other brands as well. The power of this study may have impeded the ability to know whether HP was subtly a better disinfecting agent. If there were a greater number of gowns tested, there is the potential that we could have demonstrated a statistically significant difference between the disinfection ability of HP and that of the other disinfectants. We noticed that while HP is low-to-nontoxic, there was mild stinging of the eyes noted during culture of this group and also during the fogging process despite the use of goggles. This may lower the willingness to disinfect and reuse gowns treated with this method. Increasing the length of venting times for HP may help but could lower the compliance in using this method if gowns are not readily available for use after disinfection. A lower concentration of HP may still be efficacious and may have lower adverse effects; this would need further evaluation. No adverse reactions were noted during the handling of HC gowns following disinfection.

Future studies evaluating the efficacy of HP and HC fogging against gowns inoculated with *Staphylococcus aureus*, *Escherichia coli*, and *Pseudomonas aeruginosa*, as well as used gowns in clinical practice, are warranted and underway. Studies evaluating the microbial bioburden on several brands of isolation gowns are also warranted.

## 5. Conclusions

This pilot study showed that disinfection with HP and HC fogging showed a marked reduction in microbial growth and a reduction in the types of microbes in similar proportion to EO, which is the gold standard in the sterilization of medical equipment and PPE. This is true in at least an uncontaminated gown scenario. Additional studies evaluating efficacy in a larger number of gowns and in a contaminated gown scenario are needed.

## Figures and Tables

**Table 1:** Pairwise comparisons of CFU counts using Dunn’s test.

Comparison	*p*-Values	
	@48H	@72H
control vs. ethylene oxide	0.0124	0.0065
control vs. hydrogen peroxide	0.0001	0.0000
control vs. hypochlorous acid	0.0007	0.0008
ethylene oxide vs. hydrogen peroxide	0.4680	0.3383
ethylene oxide vs. hypochlorous acid	1.0000	1.0000
hydrogen peroxide vs. hypochlorous acid	1.0000	0.9512

**Table 2:** Pairwise comparisons of numbers of microbe types.

Comparison	*p*-Values
control vs. ethylene oxide	0.0006
control vs. hydrogen peroxide	<0.0001
control vs. hypochlorous acid	0.0001
ethylene oxide vs. hydrogen peroxide	0.0669
ethylene oxide vs. hypochlorous acid	0.3188
hydrogen peroxide vs. hypochlorous acid	0.1520

**Table 3:** Fogging times for 5 randomly selected gowns in the hydrogen peroxide (HP) and hypochlorous acid (HC) groups. The randomly selected gown numbers are listed in the table in addition to the disinfection method and fogging times.

Group	Gown #	Fogging Time (min: s)
HP	1	1:15
HP	6	1:14
HP	7	1:11
HP	8	1:07
HP	9	1:05
HC	1	1:14
HC	2	1:03
HC	7	1:09
HC	9	1:13
HC	10	1:16

## Data Availability

The original contributions presented in this study are included in the article. Further inquiries can be directed to the corresponding author.

## Abbreviations

The following abbreviations are used in this manuscript:

PPE	Personal protective equipment
MNP	Micro- and nanoplastic
PPM	Parts per million
HP	Hydrogen peroxide
HC	Hypochlorous acid
EO	Ethylene oxide
CFU	Colony-forming unit
